# Surgical management of gastroesophageal reflux disease after sleeve gastrectomy with satisfactory weight loss: a SOFFCO-MM practice survey

**DOI:** 10.1007/s00423-026-04063-w

**Published:** 2026-05-20

**Authors:** Francesco Abboretti, Tigran Poghosyan, Styliani Mantziari, François Pattou, Andrea Lazzati, Marie-Cécile Blanchet, Claire Blanchard, Jean-Marc Catheline, Laurent Genser, Fabian Reche, Maud Robert, Caroline Gronnier, Damien Bouriez

**Affiliations:** 1 Department of Visceral Surgery, Lausanne University Hospital (CHUV), Lausanne, Switzerland; 2 Faculty of Biology and Medicine, University of Lausanne (UNIL), Lausanne, Switzerland; 3 Université Paris Cité, AP-HP. Nord, Hôpital Bichat Claude Bernard, Service de Chirurgie Digestive UMR 1149, Inserm, Paris, France; 4 Service de chirurgie générale et endocrinienne, CHU de Lille, Lille, France; 5 Inserm, Lille Pasteur Institute, EGID, U1190, université Lille, CHU Lille, Lille, France; 6 Department of Digestive, Bariatric and Endocrine Surgery, HU Paris Seine-Saint-Denis, AP-HP, Avicenne Hospital, Bobigny, France; 7 HeKA INSERM, INRIA Paris, Centre de Recherche des Cordeliers Paris, Université Paris Cité, Paris, France; 8 Centre Lyonnais de chirurgie digestive, CSO Sauvegarde Lyon, Lyon, France; 9 Chirurgie cancérologique, digestive et endocrinienne, institut des maladies de l’appareil digestif (IMAD), CHU de Nantes, Nantes, France; 10 CHU de Nantes, l’institut du thorax, Nantes université, CNRS, Inserm, Nantes, France; 11 Service de chirurgie digestive, centre hospitalier de Saint-Denis, Saint- Denis, France; 12 Sorbonne Université, Assistance Publique-Hôpitaux de Paris, AP-HP, Department of Hepato-Biliary and Pancreatic Surgery, Pitié-Salpêtrière University Hospital, 47-83 boulevard de l’Hôpital, Paris, 75013 France; 13 Univ. Grenoble Alpes, CNRS, UMR 5525, VetAgro Sup, Grenoble INP, INSERM, TIMC, Grenoble, 38000 France; 14 Department of Digestive Surgery, CHU Grenoble Alpes, Grenoble, France; 15 Service de chirurgie digestive et bariatrique, hospices Civils de Lyon, hôpital Edouard-Herriot, Lyon, France; 16 Esophagogastric Surgery Unit, Haut Lévêque Hospital, Centre Hospitalier Universitaire Bordeaux, France, University of Bordeaux, Bordeaux, France

**Keywords:** Gastroesophageal reflux disease (GERD), Sleeve gastrectomy (SG), Roux-en-Y gastric bypass (RYGB), Satisfactory weight loss, Metabolic bariatric surgery

## Abstract

**Introduction:**

Sleeve gastrectomy (SG) has become the most frequent bariatric surgery procedure. Secondary gastroesophageal reflux (GERD) may indicate conversion to Roux-en-Y gastric bypass (RYGB). However, in patients with sufficient weight loss, there is no clear answer in the literature as to whether RYGB should be performed with loop lengths leading to metabolic effect, or whether it should be performed solely for anti-reflux purposes. We aimed to evaluate current surgical practices and limb-length preferences among French-speaking bariatric surgeons managing GERD after SG in patients with satisfactory weight loss.

**Methods:**

A cross-sectional survey was distributed to members of the Société Française et Francophone de Chirurgie de l'Obésité et des Maladies Métaboliques (SOFFCO-MM). The questionnaire included demographics, criteria for “satisfactory weight loss”, diagnostic workup for GERD and RYGB technical configurations.

**Results:**

Fifty-three surgeons responded. Only 22.6% reported using a “standard” French RYGB configuration: alimentary limb (AL) 140-150 cm, biliary limb (BL) 70-80 cm). Most surgeons (60%) do not adjust the AL length based on weight loss, while 74% do not adjust the BL. Diagnostic approaches varied: 40% routinely performed pH monitoring, and only three surgeons required manometry.

**Conclusions:**

While the majority of surgeons do choose RYGB to manage GERD after SG in patients with satisfactory weight loss, only a minority follow a strictly “standard” configuration (AL 140-150 cm, BL 70-80 cm). This indicates notable variability in limb-length selection, underscoring the need for more harmonized approaches and clearer guidelines.

**Supplementary Information:**

The online version contains supplementary material available at https://doi.org/10.1007/s00423-026-04063-w.

## Highlights

Most surgeons don’t perform classic RYGB for GERD post SG and satisfactory weight loss.

Significant variability exists in limb lengths used during RYGB.

Clear international guidelines for RYGB limb lengths are currently lacking.

Further research is needed to optimize GERD treatment post-SG.

**Supplementary Information:**

The online version contains supplementary material available at https://doi.org/10.1007/s00423-026-04063-w.

## Introduction

Among the various bariatric surgery procedures, Sleeve Gastrectomy (SG) has emerged as one of the safest and most frequently performed, associated with favorable long-term weight loss outcomes [1, 2]. Nevertheless, SG can exacerbate or induce gastroesophageal reflux disease (GERD), posing a significant clinical challenge [3–5]. The incidence of GERD following a SG procedure is estimated to be between 19% and 30% [6–8]. Moreover, rates of de novo postoperatively GERD have been reported in more than 40% of patients [9]. In several postoperative endoscopic studies, an incidence rate of Barrett’s esophagus at 8–18.8% has been reported [8, 10], although there is still no evidence linking SG to an increased risk of esophageal cancer [11]. Along with unsatisfactory weight loss, GERD represents one of the primary indications for surgical revision following SG. A meta-analysis has identified a revisional rate of 19.9%, with 2.9% attributed specifically to GERD [12] while in other studies, the revision rate varies between 11% and 50% [4, 6, 13]. The American Society for Metabolic and Bariatric Surgery Revision Task Force recommends that the initial treatment should consist of medical therapy, with conversion to Roux-en-Y Gastric Bypass (RYGB) considered in cases of refractory symptoms or anatomical etiology for post-SG GERD [14]. For suitable candidates for RYGB, two distinct scenarios can be identified. First, the patient may have lost insufficient weight (or regained weight), necessitating a surgical revision that addresses obesity as a second-line treatment. In such cases if a RYGB is chosen as second line revision procedure, it will typically be a “standard” RYGB (as commonly performed in French practice) with an alimentary limb (AL) of 140–150 cm and a biliary limb (BL) of 70–80 cm [15, 16]. Alternatively, if the patient has achieved satisfactory weight loss (often characterized as excess weight loss (EWL) > 50% or total weight loss (TWL) > 20% [17]), the intervention’s primary purpose is to treat GERD. In this context, practices vary between “standard” RYGB, “shorter” RYGB (AL < 100 cm and < 50 cm BL) or intermediate configurations (AL between 100 and 140 cm, BL between 50 and 70 cm). These categories were operationally defined for this survey. Also, the limb length could be adapted to weight loss or comorbidities resolution. Several studies have demonstrated that a longer biliary limb is associated with greater weight loss [18]. Conversely, other meta-analyses have shown no significant differences in terms of BMI reduction and resolution of comorbidities between long versus short biliary limbs [19]. This discrepancy highlights the complexity of determining the optimal limb length in bariatric surgery and suggests that individual patient factors may play a crucial role in surgical outcomes. In this subset, it is crucial to consider that improper treatment can potentially lead to significant risks, including weight regain or nutritional deficiencies [20, 21]. The primary objective of our survey among SOFFCO-MM (“*Société Française et Francophone de Chirurgie de l’Obésité et des Maladies Métaboliques*”) members in French-speaking countries is to elucidate current practices and perspectives on managing GERD post-SG in patients with satisfactory weight loss, with a particular focus on the technical preferences for RYGB configurations involving standard versus shorter biliary and alimentary limbs.

## Methods

### Study design and participants

This cross-sectional survey was conducted among French-speaking bariatric surgeons who are members of the SOFFCO-MM (France, Switzerland). The list of participants is available in Supplementary Material 1. Eligibility for participation was limited to all surgeons actively practicing bariatric surgery and registered as members of SOFFCO-MM (total of 323 members). The association gave its approval for the distribution of the survey invitation. This article does not contain any studies with human participants or animals performed by any of the authors.

### Survey development and administration

The survey consisted of both multiple-choice and open-ended questions. Survey items were developed by the study team based on a targeted review of the available literature and current practice recommendations, and refined through internal discussions to ensure relevance and comprehensiveness. The questionnaire was not formally pilot-tested or cognitively pretested prior to dissemination. The Google Forms platform was used to administer the survey, which was distributed via email on March 3, 2023, and remained open until May 15, 2023. To increase response rates, reminders were sent bi-weekly during the survey period. Specifically, it contained 28 items addressing four main areas: (1) center and surgeon case volume, (2) thresholds used to define “satisfactory” weight loss (question wording: “From which excess weight loss (EWL) threshold do you consider the result satisfactory? i.e., not requiring the revisional procedure to provide any additional weight-loss effect”), (3) diagnostic workup for GERD after SG, and (4) surgical preferences for revision (e.g., limb lengths). The survey queries are available in Supplementary Material 2 (in French).

### Data collection

The questionnaire solicited information on demographic data, types and numbers of surgical procedures performed, decision-making processes and preferences for managing GERD in patients with or without satisfactory weight loss post-SG. Key outcomes included each surgeon’s definition of “satisfactory weight loss,” the diagnostic approach for GERD, and the specific surgical techniques used for revision (such as limb lengths). Participants could choose to skip questions or provide additional comments where they felt necessary.

### Statistical analysis

Descriptive statistics were employed to summarize the data, with categorical variables presented as percentages and continuous variables as means and standard deviations or medians with interquartile ranges, depending on data distribution. The analysis was conducted using Microsoft Excel (Microsoft Corporation), 95% binomial confidence intervals were calculated using the Wilson score method in R (R Foundation for Statistical Computing, Vienna, Austria).

## Results

### Survey participation and demographics

A total of 53 bariatric surgeons from 53 different centers (52 in France and 1 in Switzerland) participated in this survey, providing a comprehensive overview of practices within the French-speaking bariatric surgery community. The response rate was 16.4% (53/323 invited members).

### Bariatric surgery volume

The annual surgery volumes reported by the centers varied significantly as showed in Table 1 (*n* = 53; 53/53 responses). About 15.1% of the centers performed fewer than 70 surgeries per year, 22.6% performed between 70 and 120 surgeries, 26.4% conducted 120 to 200 surgeries, 17% carried out 200 to 300 surgeries, and another 17% exceeded 300 surgeries annually. Similarly, individual surgeons reported a range of annual surgery volumes (*n* = 53; 53/53 responses). Approximately 37.7% of surgeons performed fewer than 70 surgeries themselves per year, 32.1% handled 70 to 120 surgeries, 16.9% undertook 120 to 200 surgeries, 9.4% managed 200 to 300 surgeries, and a small fraction of 3.8% performed over 300 surgeries (*n* = 53; 53/53 responses).

**Table 1:** Survey participation and distribution of bariatric procedures per center

Number of procedures	*N* (%)
< 70	8/53 (15.1%)
70–120	12/53 (22.6%)
120–200	14/53 (26.4%)
200–300	9/53 (17%)
> 300	9/53 (17%)
No response	1/53 (1.9%)

### Conversions from SG to RYGB

Regarding specific surgical procedures, the number of conversions from SG to RYGB, for any indication, varied across centers (52/53 responses, 1 missing). About 19.2% of centers performed fewer than 8 conversions per year, 25% performed 8 to 11 conversions, 26.9% carried out 11 to 20 conversions, 13.4% conducted 20 to 40 conversions, and 15.3% completed more than 40 conversions annually. Focusing on conversions for GERD management in patients with satisfactory weight loss post-SG (50/53 responses, 3 missing), 32% of centers reported performing fewer than 3 conversions per year for GERD alone, 34% did 3 to 10 conversions, 26% handled 10 to 20 conversions, and 6% performed more than 20 conversions annually. On an individual level, surgeons reported performing fewer conversions for GERD compared to their total annual surgeries (49/53 responses, 4 missing). About 42.8% of surgeons performed fewer than 3 conversions per year, 40.8% carried out 3 to 10 conversions, 12.2% did 10 to 20 conversions, and a minimal 4.1% performed more than 20 conversions for GERD alone each year.

### Perception of satisfactory weight loss (% EWL)

Surveyed surgeons have varying thresholds for considering weight loss post-SG as satisfactory without the need for further surgical intervention for weight loss (52/53 responses, 1 missing). Half of the respondents (50%) set this threshold at 50% EWL, with a significant number considering 60% EWL (21%) and 70% EWL (8%) as satisfactory. Notably, a smaller percentage of surgeons (6%) considered 80% EWL as the benchmark for satisfactory weight loss.

### Diagnostic approach to GERD after SG

When confronted with a patient exhibiting typical clinical signs of GERD after SG (53/53), 40% (21/53; 95% CI 27.6–53.1) of surgeons reported systematically performing pH monitoring, while another 40% (21/53; 95% CI 27.6–53.1) never used this test, 20% (11/53; 95% CI 12–33.5) reserving it only for cases without esophagitis on endoscopy. Only 3/53 (95% CI 7.9–27.1) surgeons deemed manometry specifically required, and barium swallow or CT scans were used in 8/53 (95% CI 7.9–27.1) centers for assessment.

### Type of surgical procedure for GERD after SG with satisfactory weight loss

In cases where patients experience disabling GERD post-SG with satisfactory weight loss, 79% (42/53 respondents) indicated a preference for RYGB procedure. A smaller proportion, 9% (5/53), selected an approach described as “minimal limb length”, which in this context refers to a short limb RYGB with shorter alimentary and biliary limbs, in memory of the technique originally described for the treatment of GERD using a Roux-en-Y alimentary limb and a biliary limb [22]. Additionally, 6% (3/53) selected a combined approach of RYGB or Hill procedure, while the same percentage opted solely for the Hill procedure (6%, 3/53).

### Length of alimentary limb

The length of the alimentary limb created during surgery varied among surgeons (Table 2), with 18% creating limbs of 60–90 cm, 31% opting for 90–120 cm, a minority of 4% for 120–140 cm, and the majority of 47% for an alimentary limb longer then 140 cm. Most surgeons (60%, 32/53) reported they do not adjust the length of the alimentary limb based on the patient’s BMI and weight loss.

**Table 2:** Length of alimentary and biliary limbs

Limb type	N (%)
Alimentary Limb	
60-90 cm	9/51 (18%)
90-120 cm	16/51 (31%)
120-140 cm	2/51 (4%)
> 140 cm	24/51 (47%)
Biliary Limb	
30-40 cm	4/51 (8%)
50-65 cm	29/51 (57%)
70-85 cm	11/51 (21%)
90-120 cm	7/51 (14%)

### Length of biliary limb

As showed in Table 2, respondents (51/53) indicated the following preferences for the length of the biliary limb: 8% use a length of 30–40 cm, a majority of 57% prefer 50–65 cm, 21% use 70–85 cm, and 14% prefer a limb of 90–120 cm. A significant majority, 74% (39/53 respondents), reported they do not adjust the length of the biliary limb based on BMI and weight loss.

### Measurement of common channel

In this section, “minimal limb length” refers to the minimum expected common channel length reported by respondents when they measure it. Most surgeons (80%, 41/52 respondents) do not measure the common channel length, with 20% (11/52) confirming they do. For those who measure the common channel, the minimum expected lengths were 100 cm (2 surgeons), 150 cm (1 surgeon), 250 cm (3 surgeons), 300 cm (4 surgeons), and 350 cm (1 surgeon).

### “Standard” vs short vs. intermediate RYGB limbs lengths

Table 3 resume the preferences among participants in terms of RYGB limbs lengths. The survey results indicate that 23% (12/53; 95% CI 13.5–35.5) of surgeons perform a “standard” RYGB, characterized by an alimentary limb (AL) of 140–150 cm and a biliary limb (BL) of 70–80 cm. A smaller group, 8% (4/53; 95% CI 3.0–17.9), perform a “short limb” RYGB, with an AL of less than 100 cm and a BL of less than 50 cm. Meanwhile, 19% (10/53; 95% CI 10.6–31.4) reported using an “intermediate” RYGB, defined by an AL between 100 and 140 cm and a BL between 50 and 70 cm.

**Table 3:** "Standard" vs short vs intermediate RYGB limbs lengths

RYGB type	Alimentary limb (cm)	Biliary limb (cm)	N (%)
“Standard”	140-150	70-80	12/53 (23%; 95% CI 13.5-35.5)
Short	< 100	< 50	4/53 (8%; 95% CI 3.0-17.9)
Intermediate	100-140	50-70	10/53 (19%; 95% CI 10.6-31.4)
Other Combinations	Various	Various	27/53 (51%; 95% CI 37.9-63.9)

Legend: *RYGB,* Roux-en-Y gastric bypass

### Future insights

The majority of respondents, accounting for 91% (48 surgeons), indicated a positive interest in participating in a prospective trial. Only a small fraction, 7% (4 surgeons), expressed no interest in such research endeavors, while 2% (1 surgeon) did not provide a response.

## Discussion

Our survey suggests that the majority of surgeons opt for a RYGB to manage GERD in patients who have achieved satisfactory weight loss after SG. Nevertheless, we observed variability in the final RYGB configuration, particularly regarding limb lengths: while most surgeons do not adjust limb lengths based on weight loss, only 23% strictly follow a “standard” RYGB (AL 140–150 cm, BL 70–80 cm), and a considerable proportion adopt intermediate, short-limb, or other customized approaches. The term “satisfactory weight loss” remains a topic of debate in bariatric metabolic surgery. Some authors, including the IFSO 2024 Consensus, propose defining satisfactory weight loss as achieving ≥ 20% Total Weight Loss (TWL), as this measure is less influenced by preoperative BMI [17]. The 2024 guidelines from the French “Haute Autorité de Santé” (HAS) define bariatric surgery failure as achieving < 10% TWL two years post-surgery [23].

Typically, the initial management of GERD following SG involves lifestyle modifications and medical therapy [24]. Conversion to RYGB is considered only as a last resort when these interventions fail [14]. Another emerging option for managing GERD post-SG is represented by devices for magnetic sphincter augmentation, which has shown promise in controlling reflux symptoms by enhancing the function of the lower esophageal sphincter [25]. A small number of surgeons in our survey opted for the Hill modified technique, which, although controversial, has shown promising results in controlling GERD symptoms after SG without major complications in a small cohort of patients [26]. Furthermore, we observed marked heterogeneity in the pre-revisional diagnostic workup (e.g. pH monitoring and manometry), which likely reflects the absence of harmonized pathways and may contribute to variability in patient selection and management. Accordingly, there is a call for deeper understanding of the mechanisms of reflux in people with obesity and the long-term outcomes of various surgical interventions [3–5, 27, 28]. In a large retrospective study comparing outcomes between primary RYGB and SG conversion to RYGB, where GERD was the most common indication for revision, Dang et al. found a higher rate of severe complications in the conversion group (5% vs. 7.2%, *p* < 0.001) [29]. Although these complication rates do not exceed the benchmark typically expected in elective secondary bariatric surgery, which is posited to be around 14.6% [30], these findings highlight the complexities associated with surgical decisions in this patient group. Furthermore, American Guidelines suggest that conversion to standard RYGB should be considered the best option for managing GERD post-SG when resistant to medical treatment [14].

As described above, we identified three groups based on limb lengths: “standard” (AL 140–150 cm, BL 70–80 cm), “short” (AL < 100 cm, BL < 50 cm), and “intermediate” (AL 100–140 cm, BL 50–70 cm) RYGB configurations. For analytical purposes, we grouped reported limb lengths into three operational categories to facilitate descriptive reporting; these cut-offs were predefined by the authors and are not standardized in the literature. Nevertheless, most surgeons participating in the survey perform various combinations of limb lengths. On the one hand, there is probably a component of “tailored surgery”, where surgeons aim to adjust limb lengths based on the individual needs of each post-SG patient with GERD (potentially to minimize malnutrition or vitamin deficiency risk). On the other hand, there is a lack of clear international guidelines on the ideal limb lengths, not only in this specific context of conversion to RYGB post-SG for GERD, but also in the broader field of primary RYGB [16]. In cases of refractory GERD post-SG with satisfactory weight loss, the new French HAS guidelines suggest a RYGB with a 70 cm alimentary limb, without providing recommendations on the length of the biliary limb [23]. In addition, very short alimentary limbs have been associated with bile reflux symptoms, which should be considered when GERD resolution is the primary goal.

In the practical landscape of surgical revision for GERD after SG with satisfactory weight loss, there emerges a dichotomy. One perspective typically argues against adding a metabolic effect, emphasizing that the surgical focus should be solely on GERD resolution. In contrast, another perspective advocates for considering the patient’s predisposition to obesity, suggesting that without addressing the metabolic component in the surgical procedure, patients are likely to regain weight, as the body “remembers” its maximum weight. Given these contrasting viewpoints, a modified version of the classic RYGB, potentially tailored to preserve the weight loss achieved while adequately managing GERD, may represent a more fitting approach in “real life” scenarios. Such a strategy could bridge the gap between the two philosophies, aiming for comprehensive patient care that addresses both GERD and the long-term risk of weight regain. Additionally, it is important to consider the patient’s preferences: some may prioritize treating GERD while hoping to reinforce weight loss, while others may seek treatment for GERD alone, fearing further weight loss. Although data on malnutrition in patients with normal BMI undergoing RYGB conversion are sparse, this remains a point of vigilance, warranting close postoperative monitoring to mitigate risks associated with malabsorption.

The most important limitation of our study lies in the varied criteria surgeons used to define “satisfactory weight loss”. While guidelines often suggest thresholds such as > 50% EWL or > 20% TWL [17], our survey specifically queried surgeons on EWL alone, revealing a wide range of cutoff values (from 50% up to 80%). This heterogeneity reduces the internal and external validity of our findings, as different centers may categorize the same patient as having achieved “satisfactory” or “unsatisfactory” weight loss based on inconsistent definitions. Additionally, some patients might still meet criteria for obesity by BMI, irrespective of their EWL percentage, which can influence the choice of surgical technique but was not captured in our survey. Beyond this, several other limitations must be considered when interpreting our results. Firstly, the data reflect common practices reported by individual surgeons and are not based on statistical analyses of patient cohorts from each center, which may limit the robustness of the findings; moreover, the questionnaire was not formally pilot-tested or cognitively pretested prior to dissemination. Secondly, the survey was limited to SOFFCO-MM members, which may not represent the practices of bariatric surgeons globally, with an overall response rate of 16.4%. While this rate is comparable to other voluntary internet-based physician surveys [31], the possibility of non-response bias cannot be excluded. Surgeons with a particular interest in GERD or revisional bariatric surgery may be overrepresented, potentially limiting the generalizability of our findings. Thirdly, the “standard”, “short”, and “intermediate” RYGB limb-length categories were operationally defined for this survey and are not standardized in the literature; therefore, apparent between-group variability should be interpreted cautiously, as it may partly reflect differences in terminology rather than clinically meaningful distinctions. Finally, the survey did not differentiate between the specific techniques and protocols used in different centers, which could impact the generalizability of the findings.

Our study points to the pressing need for additional research. An overwhelming 91% of respondents in our survey expressed interest in participating in prospective studies. This demonstrates a collective eagerness for empirical research that could guide the refinement of surgical techniques and optimize patient-centered outcomes in the treatment of GERD following SG.

## Conclusion

In patients with disabling GERD after SG and satisfactory weight loss who are resistant to medical therapy, our survey indicates that conversion to RYGB is the most commonly preferred revisional strategy among responding surgeons. The results reveal significant variability in the limb lengths configurations used for RYGB in this setting. This heterogeneity supports the need for more standardized pathways and prospective outcome studies to identify which technical variations are clinically meaningful while minimizing the risks of malnutrition and weight regain.

## Supplementary Information

Below is the link to the electronic supplementary material.

Supplementary Material 1

Supplementary Material 2

## Data Availability

No datasets were generated or analysed during the current study.
