# Supplementary material for: Surgical management of gastroesophageal reflux disease after sleeve gastrectomy with satisfactory weight loss: a SOFFCO-MM practice survey
Source: Langenbecks Arch Surg. 2026 May 20;411(1):283. doi: 10.1007/s00423-026-04063-w (PMC13672564; doi:10.1007/s00423-026-04063-w)
Supplement: Supplementary file 1 [file 423_2026_4063_MOESM2_ESM.pdf]

Enquête de pratique de la SOFFCO-MM : Conversion de Sleeve en Bypass pour reflux gastro-oesophagien chez le patient ayant une perte de poids satisfaisante

Le Bureau de la SOFFCO-MM s'intéresse aux pratiques des chirurgiens bariatriques français en cas de conversion de Sleeve en Gastric-bypass en Y (GBP) pour reflux gastro-oesophagien (RGO) avec perte de poids satisfaisante.
Plusieurs stratégies ont été identifiées face à cette situation (GBP "classique", diversion duodénale totale ou GBP sur mesure avec des longueurs d'anses adaptées à la perte de poids)

Les conversions de Sleeve en GBP pour twist et pour fistule chronique ne sont pas traitées dans cette enquête.

1. Quel est votre nom ?

2. Nom de votre centre ?

3. Approximativement, combien d'interventions bariatriques sont réalisées par an dans **votre centre** ?

4. Approximativement, combien d'interventions bariatriques **réalisez vous vous-même** par an ?

5. Approximativement, combien de conversion de Sleeve en Gastric-bypass en Y (**quelque soit l'indication**) sont réalisées dans **votre centre** par an ?

6. Approximativement, combien de conversion de Sleeve en Gastric-bypass en Y (**quelque soit l'indication**) réalisez vous vous-même par an ?

7. Approximativement, combien de conversion de Sleeve en GBP (pour **perte de poids insuffisante** sans RGO) sont réalisées dans **votre centre** par an ?

8. Approximativement, combien de conversion de Sleeve en Gastric-bypass en Y (pour **perte de poids insuffisante** sans RGO) réalisez vous **vous-même** par an ?

9. Approximativement, combien de conversion de Sleeve en Gastric-bypass en Y (pour **perte de poids insuffisante et RGO**) sont réalisées dans **votre centre** par an ?

10. Approximativement, combien de conversion de Sleeve en Gastric-bypass en Y (pour **perte de poids insuffisante et RGO**) réalisez vous **vous-même** par an ?

11. Approximativement, combien de conversion de Sleeve en Gastric-bypass en Y (**pour RGO chez des malades qui ont une perte de poids satisfaisante**) sont réalisées dans **votre centre** par an ?

____

12. Approximativement, combien de conversion de Sleeve en Gastric-bypass en Y (**pour RGO chez des malades qui ont une perte de poids satisfaisante**) réalisez vous **vous-même** par an ?

____

13. A partir de quelle **perte d'excès de poids** considérez vous le résultat satisfaisant (ne nécessitant pas que le nouveau montage ait un effet supplémentaire sur la perte de poids) ?

Une seule réponse possible.

- ( ) >20%
- ( ) >30%
- ( ) >40%
- ( ) >50%
- ( ) >60%
- ( ) >70%
- ( ) >80%
- ( ) >90%
- ( ) >100%
- ( ) Autre : _______________________________

14. Devant un patient ayant un RGO avec signes cliniques typiques après Sleeve, quel bilan réalisez vous ? (plusieurs réponses possibles)

Plusieurs réponses possibles.

- [ ] Vous faites une fibroscopie oesogastroduodénale
- [ ] Vous faites une fibroscopie et pHmetrie systématique
- [ ] Vous faites la pHmetrie que si l'oesophage est normal ou si l'oesophagite est de grade A ou B
- [ ] Vous faites une manometrie oesophagienne systématiquement
- [ ] Autre : ______________________

15. Quelles sont vos indications de conversion de Sleeve en Gastric-bypass en Y pour RGO ?

Plusieurs réponses possibles.

- [ ] Endobrachyoesophage à la fibroscopie systématique
- [ ] Oesophagite à la fibroscopie systématique
- [ ] RGO symptomatique
- [ ] Autre : ______________________

16. Chez un patient ayant eu une Sleeve avec une perte d'excès de poids considérée comme satisfaisante et un RGO invalidant pour lequel vous posez une indication opératoire, quelle intervention réalisez-vous ?

Une seule réponse possible.

- ( ) Un Gastric-bypass en Y
- ( ) Une intervention de Hill
- ( ) Une diversion duodénale
- ( ) Autre : ______________________

17. Quelle voie d'abord utilisez vous ?

Plusieurs réponses possibles.

- [ ] Laparotomie
- [ ] Coelioscopie
- [ ] Coelioscopie avec assistance du robot

18. Combien mesure l'anse alimentaire que vous réalisez (en cm)?

____

19. Adaptez vous la longueur de l'anse alimentaire à la perte de poids ?

Une seule réponse possible.

- ( ) Oui
- ( ) Non

20. Si oui, comment ?

____

21. Combien mesure l'anse biliaire que vous réalisez (en cm) ?

____

22. Adaptez vous la longueur de l'anse biliaire à la perte de poids ?

Une seule réponse possible.

- ( ) Oui
- ( ) Non

23. Si oui, comment ?

____

24. Mesurez vous l'anse commune ?

Une seule réponse possible.

- ( ) Oui
- ( ) Non

25. Si oui, quelle longueur minimale est attendue (en cm)?

____

26. Réalisez vous une vagotomie tronculaire ?

Une seule réponse possible.

- ( ) Toujours
- ( ) Jamais
- ( ) Autre : ____________________

27. Seriez-vous intéressé de participer à une étude prospective sur le sujet ?

Une seule réponse possible.

- ( ) Oui
- ( ) Non

28. Avez vous des remarques ou commentaires sur la prise en charge chirurgicale du RGO après Sleeve

Ce contenu n'est ni rédigé, ni cautionné par Google.

Google Forms
