# Supplementary material for: Surgical management of gastroesophageal reflux disease after sleeve gastrectomy with satisfactory weight loss: a SOFFCO-MM practice survey
Source: Langenbecks Arch Surg. 2026 May 20;411(1):283. doi: 10.1007/s00423-026-04063-w (PMC13672564; doi:10.1007/s00423-026-04063-w)
Supplement: Supplementary file 2 [file 423_2026_4063_MOESM1_ESM.docx]

Original article to be submitted to *Langenbeck's Archives of Surgery*

**Surgical Management of Gastroesophageal Reflux Disease after Sleeve Gastrectomy with Satisfactory Weight Loss: What do Surgeons do? A SOFFCO-MM Practice Survey in France.**

Supplementary material 1 - List of participants

1. **Dr. Olivier Fercocq**, Clinique du Ter, Ploemeur, France
2. **Dr Arnaud Liagre**, Clinique des Cedres, Cornebarrieu, France
3. **Dr. Adel Abou-Mrad**, CHR Orléans, Orléans, France
4. **Dr. Alice Cenedese**, Centre de Chirurgie Viscérale et Digestive du Nord Isère, Clinique Saint Vincent de Paul, Bourgoin-Jallieu, France.
5. **Dr. Nathan Beaupel**, Clinique de Bercy, Charenton-le-Pont, France.
6. **Dr. Amine Bouayed**, Centre Hospitalier de Salon-de-Provence, Salon-de-Provence, France.
7. **Dr. Lauren Arnalsteen**, Hôpital Privé La Louvière, Lille, France.
8. **Dr. Salomon Benchetrit**, Clinique du Parc, Lyon, France.
9. **Dr. Emeric Abet**, Centre Hospitalier Départemental Vendée, La Roche-sur-Yon, France.
10. **Dr. Belhassen Seket**, Centre Hospitalier de la Région de Saint-Omer, Saint-Omer, France.
11. **Dr. Jean Marc Catheline**, Centre Hospitalier de Saint-Denis, Saint-Denis, France.
12. **Dr. Jean François Ain**, Polyclinique du Val de Saône, Mâcon, France.
13. **Dr. Henry Mercoli**, Polyclinique Franche-Comté, Besançon, France.
14. **Dr. Fanelly Torres**, Cabinet Nemaudig, Nîmes, France.
15. **Dr. Chakib Abbadi**, Clinique de l'Yvette, Longjumeau, France.
16. **Dr. Litavan Khamphommala**, CHP Saint-Grégoire, Saint-Grégoire, France.
17. **Dr. Géraud Tuyeras** CHU Toulouse, Toulouse, France.
18. **Dr. Pascal Jagot-Lacoussiere,** Clinique du Pré, Le Mans, France.
19. **Dr. Nelson Trelles**, Centre Hospitalier NOVO, Pontoise, France.
20. **Dr. Christophe Cazères**, Centre de l’Obésité de la Clinique des Orchidées, La Réunion, France.
21. **Dr. Andre Dabrowski**, Clinique de Saint-Omer et GHICL Lomme, Saint-Omer/Lomme, France.
22. **Pr. François Pattou**, CHU Lille, Lille, France.
23. **Dr. Sergio Carandina**, Centre Chirurgical de l'Obésité, Toulon, France.
24. **Dr. Sophie Al Samman**, Infirmerie Protestante, Lyon, France.
25. **Dr. Thierry Bégué**, CHU Nord Marseille APHM, Marseille, France.
26. **Dr. Jean-Luc Luciani**, Polyclinique Maymard, Bastia, France.
27. **Dr. Guillaume Pourcher**, Institut Mutualiste Montsouris, Paris, France.
28. **Dr. Philippe Lasserre**, Clinique Occitanie, Muret, France.
29. **Dr. Francis Genier**, Clinique Jules Verne, Nantes, France.
30. **Dr. Alexandre Filippello**, Hôpital Privé de la Loire, Saint-Étienne, France.
31. **Dr. Michel Vix**, CHRU Strasbourg, Strasbourg, France.
32. **Dr. Antoine Soprani**, Clinique Geoffroy Saint-Hilaire, Paris, France.
33. **Dr. Gerold Schroder Gerold**, Clinique des Landes, Mont-de-Marsan, France.
34. **Dr. Stéphane Servajean**, Clinique du Parc Monceau et Clinique de l'Estrée, Paris/Stains, France.
35. **Dr. Salim Jabahgi**, Centre Hospitalier Marie Madeleine, Forbach, France.
36. **Dr. Ludovic Billard**, Hôpital Privé Dijon Bourgogne, Dijon, France.
37. **Dr. Rachid Bousfiha**, Hôpital de Compiègne, Compiègne, France.
38. **Dr. Philippe Keller**, Hôpitaux Civils de Colmar, Colmar, France.
39. **Dr. Laurent Passebois**, Centre Hospitalier de Béziers, Béziers, France.
40. **Dr. Luigi De Magistris,** Centre Hospitalier de Mâcon, Mâcon, France.
41. **Dr. Cristian Bivoleanu**, Polyclinique du Val de Sambre, Maubeuge, France.
42. **Dr. Eva Laurent**, Clinique du Tondu Florian, Bordeaux, France.
43. **Dr. Ion Donici**, CHU Nîmes, Nîmes, France.
44. **Dr. Claire Philippe**, Clinique Générale, Annecy, France.
45. **Dr. Natalia Chilintseva**, Clinique Sainte Odile, Haguenau, France.
46. **Dr. Daniel Lazescu**, Clinique du Vivarais, Aubenas, France.
47. **Dr. Milan Smejkal**, Clinique du Pays de Rance, Dinan, France.
48. **Dr. Eric Boulay**, Clinique Sainte Thérèse, Paris, France.
49. **Dr. Dorothée Brachet**, Pôle Santé Sud Le Mans, Le Mans, France.
50. **Dr. Thomas Gautier**, Clinique Saint Jean, Montpellier, France.
51. **Dr. Damien Bouriez**, CHU Bordeaux, Bordeaux, France.
52. **Dr. Styliani Mantziari**, CHUV Lausanne, Lausanne, Switzerland.
53. **Dr. Jean-Louis Lorin**, Clinique La Parisière, Bourg-de-Péage, France.
